# The Duration of Life after the Appearance of Albuminuric Retinitis

**Published:** 1905-08-05

**Authors:** 


### THE DURATION OF LIFE AFTER THE APPEARANCE OF ALBUMINURIC RETINITIS.

MR. SIMEON SNELL[1] considers that the duration of life after the appearance of retinitis in cases of albuminuria is less in hospital than in private practice. Private patients with their better financial position are able to attend to the dietetic and hygienic treatment demanded in Bright's disease, and in hospital patients, further, the influence of alcoholism has probably a greater range. The statistics collected by Dr. G. O. Belt, of Washington, show that in a large collection of cases 62 per cent. of the private patients died in the first year and 14 per cent. lived more than two years. In hospital cases, on the other hand, 85 per cent. died in the first year and only 6 per cent. lived more than two years. Mr. Snell qotes a series of private cases in which the average duration of life was just under a year. This contrasts with a series of hospital cases observed by Dr. Miles Miley with an average length of survival of four-and-a-half months. In the former group there were several instances of survival for two to three years and in one case death was postponed for four-and-a-half years. These figures do not refer to albuminuric retinitis occurring during the course of pregnancy.

[1] Lancet, July 15, 1905.

